# Promoter Sequences Do Not Solely Govern *nosZ* Expression Differences between *Bradyrhizobium ottawaense* and *B. diazoefficiens*

**DOI:** 10.1264/jsme2.ME25079

**Published:** 2026-03-03

**Authors:** Sawa Wasai-Hara, Yoshikazu Shimoda, Hisayuki Mitsui, Shusei Sato, Haruko Imaizumi-Anraku, Kiwamu Minamisawa

**Affiliations:** 1 National Agriculture and Food Research Organization (NARO), Kannondai, Tsukuba, Ibaraki 305–8604, Japan; 2 National Institute of Advanced Industrial Science and Technology (AIST) Hokkaido, 2–17–2–1 Tsukisamu-higashi, Toyohira, Sapporo, Hokkaido 062–8517, Japan; 3 Graduate School of Life Sciences, Tohoku University, Katahira, Aoba-ku, Sendai 980–8577, Japan

**Keywords:** N_2_O reduction, greenhouse gases, gene expression, rhizobia, promoter sequence

## Abstract

Nitrous oxide (N_2_O) is a potent greenhouse gas, and the enzyme Nos catalyzes its reduction to dinitrogen (N_2_). *Bradyrhizobium ottawaense* exhibits strong N_2_O-reducing activity with high *nosZ* expression. To investigate whether promoter sequences affect *nosZ* expression, we constructed reciprocal promoter-swapped mutants between *B. ottawaense* and *B. diazoefficiens*. The swapping of promoters did not significantly affect expression levels. *B. ottawaense* mutants maintained approximately 200-fold higher expression levels than *B. diazoefficiens*, and the introduction of the *B. ottawaense* promoter into *B. diazoefficiens* did not increase expression levels. Therefore, the present results indicate that promoter sequence differences are not the primary factor affecting *nosZ* expression, suggesting regulation by other factors.

Nitrous oxide (N_2_O) is a long-lived atmospheric gas that acts both as a potent greenhouse gas, with a global warming potential that is approximately 300-fold that of CO_2_, and as an ozone-depleting substance (Ravishankara *et al.*, 2009; IPCC, 2023). Its atmospheric concentration has risen by >20% since the pre-industrial era, mainly due to agriculture, which accounts for more than half of anthropogenic emissions (Tian *et al.*, 2020). Therefore, reducing N_2_O emissions from croplands is a critical challenge in the mitigation of climate change.

N_2_O reduction occurs in some bacteria and archaea that possess nitrous oxide reductase (NosZ) (Hallin *et al.*, 2018). Since the 1980s, *nosZ* genes and their enzymatic activities have been characterized (Bhandari and Nicholas, 1984). More recently, the discovery of novel *nosZ* clades, Clades II and III (Sanford *et al.*, 2012; He *et al.*, 2025), has expanded our understanding of microbial N_2_O reduction and opened up possibilities for agricultural mitigation technologies using N_2_O-reducing bacteria (Itakura *et al.*, 2013; Hiis *et al.*, 2024; Nishida *et al.*, 2025).

Several rhizobia, including *Bradyrhizobium diazoefficiens*, harbor the classical Clade I *nosZ* and are capable of growing with N_2_O as the sole electron acceptor (Sameshima-Saito *et al.*, 2006; Wasai-Hara *et al.*, 2023). Field applications using a *nosZ*-overexpressing strain of *B. diazoefficiens* have demonstrated the potential for “microbial N_2_O mitigation” (Itakura *et al.*, 2013). However, wild-type strains exhibit insufficient N_2_O-reducing activity, making naturally occurring high-activity strains desirable for practical applications.

The expression of the *nos* operon is regulated by FixLJK_2_ and RegSR in response to low oxygen levels (Torres *et al.*, 2017; Sciotti *et al.*, 2003), and by NasST in response to the presence of nitrate (Sánchez *et al.*, 2014, 2017). A recent study suggested that when nitrate (NO_3_^–^) and N_2_O are both present, N_2_O is preferentially reduced, highlighting the role of electron allocation and the possible involvement of novel transcriptional regulatory mechanisms (Gao *et al.*, 2021).

We previously isolated the rhizobial strain, *Bradyrhizobium ottawaense*, and found that its N_2_O-reducing activity was seven- to eight-fold higher than that of *B. diazoefficiens* (Wasai-Hara *et al.*, 2020, 2023). This high activity is considered to be attributable to the up-regulated expression of *nosZ* (Wasai-Hara *et al.*, 2023). Furthermore, sequence anal­yses and mutant-based experiments suggested that known regulatory systems (FixLJK_2_, RegSR, and NasST) are not responsible for this up-regulated expression. Low sequence conservation (48% homology) has been observed in the promoter sequence upstream of *nosZ*. In addition, two transcription start sites were detected in *B. ottawaense* under N_2_O-respiring conditions, whereas *B. diazoefficiens* possessed only a single start site, indicating that sequence variations in this region affect the transcriptional levels of the *nos* operon (Wasai-Hara *et al.*, 2023).

We herein exami­ned the contribution of promoter sequences to *nosZ* expression by constructing reciprocal promoter-swapped mutants between *B. ottawaense* SG09 (high activity) and *B. diazoefficiens* USDA110^T^ (low activity). We compared *nosZ* expression levels in these mutants to investigate whether promoter differences explain the up-regulated expression observed in *B. ottawaense*.

The construction of promoter-swapped mutants is described below. The promoter including the region and untranslated region (UTR), as defined in the present study, are shown in Fig. 1A and B. Transcription start sites were previously identified (P_d1_ and P_d2_), corresponding to the two‍ ‍promoter-including regions (Promoter #1/#2) in *B. ottawaense* SG09. Mutants, in which only the promoter sequence or both the promoter and UTR sequences were replaced, were constructed by first deleting the 286 bp (80+133+73 bp) upstream of the transcription start site of SG09 using the in-frame markerless deletion method (Fig. 1C) (Wasai-Hara *et al.*, 2023). The up- and downstream regions of the promoter and UTR region were amplified with the primers listed in Table S1 and S2 and with Prime STAR^®^ Max DNA Polymerase (Takara Bio). The fragments were combined by overlap extension PCR and cloned into the *Sma*I site of the suicide vector pK18mobsacB-Ω using an In-fusion HD Cloning Kit (Takara Bio). The sequences of the introduced fragments were confirmed by sequencing. Transmission of the plasmid to *B. ottawaense* SG09 and homologous recombination of the promoter region were achieved by triparental mating. Transconjugants were selected based on resistance to streptomycin (100‍ ‍μg mL^–1^), spectinomycin (100‍ ‍μg mL^–1^), and polymyxin (100‍ ‍μg mL^–1^) and sensitivity to 10% sucrose. Single crossover strains were then cultured in HM medium without antibiotics, and deletion mutants—lacking the promoter and/or UTR and exhibiting sensitivity to Sp/Sm and resistance to sucrose—were obtained. The alternative promoter (80 bp) and UTR (83 bp) from *B. diazoefficiens* USDA110^T^ were then inserted into the genome at the original locus (ΔP_nos_::110, ΔP_nos_::110+UTR) using the same in-frame markerless method. The validity of mutant constructs was confirmed by DNA sequencing. Additional mutants lacking either of the two promoters were also generated (ΔP1 and ΔP2). To assess the effects of upstream sequences, a partial ferritin deletion mutant (Δftn _[Δ763+IGR488]_) was constructed, retaining the promoter, but lacking the upstream ferritin and intergenic regions (Fig. 1C).

In *B. diazoefficiens* USDA110^T^, mutants with swapped promoter regions alone, swapped promoter and UTR regions together, and promoter deletion mutants were constructed. Furthermore, a deletion mutant encompassing part of the ferritin gene and the upstream intergenic region (Δftn_[Δ877+IGR99]_) was generated (Fig. 1C).

*nosZ* gene expression levels were measured by RT-qPCR as previously described (Wasai-Hara *et al.*, 2023). *B. ottawaense* SG09, *B. diazoefficiens* USDA110^T^, and their mutant lines were cultured in 10‍ ‍mL of HM medium (Cole and Elkan, 1973) in 75-mL glass tubes at 28°C with shaking at 180‍ ‍rpm. The headspace was replaced with N_2_, and 3.25‍ ‍mL of 100% N_2_O was added as the sole electron acceptor, resulting in a final concentration of 5% (Wasai-Hara *et al.*, 2023). Pre-cultured cells were inoculated into the HM medium at an initial OD_660_=0.05, and total RNA was extracted after overnight cultivation (final OD reached approximately 2.0±0.5). Fold changes in gene expression were calculated using the ΔΔC_t_ method. Raw C_t_ values were initially normalized to the internal reference gene (*sigA*), and the fold change from the control condition was then calculated.

The *nosZ* transcript level in *B. ottawaense* SG09 wild-type was 220±37-fold higher than that in *B. diazoefficiens* USDA110^T^ (Fig. 2A and S2A), consistent with previous findings (Wasai-Hara *et al.*, 2023). The replacement of the *nosZ* promoter in SG09 with that from USDA110^T^ (SG09ΔP_nos_::110) did not significantly affect expression levels, which remained 279±60-fold higher. Therefore, promoter replacement was considered to not affect *nosZ* expression levels. In contrast, swapping of both the promoter and UTR sequences (SG09ΔP_nos_::110+UTR) resulted in a significant reduction in *nosZ* expression (Fig. 2A). This may attributed to the mRNA of the UTR region in USDA110^T^ forming a hairpin secondary structure under nitrate-free conditions, which may sterically hinder the access of transcription machinery and thereby inhibit transcription (Sánchez *et al.*, 2017). Therefore, the introduction of the USDA110^T^-derived UTR sequence in this study was considered to have inhibited transcription to a similar extent. The negative effects of the UTR were attenuated under nitrate-respiring conditions (Fig. S1 and S2B)

The deletion of either promoter individually (SG09ΔP1 and ΔP2) resulted in a significant decrease in *nosZ* expression (Fig. 2A). Therefore, both promoters in SG09 were functionally active under N_2_O-respiring conditions. In contrast, the deletion of an upstream region (SG09Δftn_[Δ763+IGR488]_) that left both promoter sequences intact did not cause a significant reduction in *nosZ* transcript levels (Fig. 2A). Based on these results, the upstream region defined in the present study was not considered to affect *nosZ* expression.

In *B. diazoefficiens* USDA110^T^, the replacement of the promoter region with that of SG09 (USDA110^T^ΔP_nos_::SG09) or with both the promoter and UTR (USDA110^T^ΔP_nos_::SG09+UTR) resulted in no significant change in expression (Fig. 2A). Similarly, the deletion of the upstream gene region (USDA110^T^Δftn_[Δ877+IGR99]_) did not affect *nosZ* expression. These results, similar to those observed for SG09, indicate that in the USDA110^T^ background, the promoter sequence itself did not affect *nosZ* expression.

Furthermore, the complete deletion of the promoter region abolished growth under N_2_O-respiring conditions in both species, although the mutants grew normally under nitrate-respiring or aerobic conditions, confirming that the presence of the promoter is indispensable for *nosZ* expression and, thus, N_2_O respiration (Fig. 2B, C, and S3).

Collectively, the present results demonstrate that the promoter region is essential for *nosZ* expression in both *B. ottawaense* SG09 and *B. diazoefficiens* USDA110^T^, whereas the promoter sequence itself does not affect expression levels. Since promoter activity is generally considered to be a key factor affecting gene expression (Hawley and McClure, 1983; Wang *et al.*, 2023), these results are unexpected and noteworthy. Heterologous promoters may be substituted with similar efficiency, indicating that differences in *nosZ* expression between the two strains are primarily governed by trans-acting factors rather than by promoter cis-elements. Such regulation may involve transcriptional activators recognizing conserved motifs within the *nos* promoter between *B. ottawaense* and *B. diazoefficiens*, or, alternatively, species-specific differences in transcriptional or translational resource allocation or in mRNA stability (Zhu and Dai, 2025). Possible approaches to identify factors that regulate expression levels in *B. ottawaense* include screening for proteins that bind to the *nos* promoter site, narrowing down candidate factors that are co-expressed with the *nos* gene through a transcriptome anal­ysis, and the genome-wide screening of regulatory elements using random transposon mutagenesis. On the other hand, it remains unclear whether high *nosZ* expression in *B. ottawaense* reflects species-specific activation or repression in *B. diazoefficiens*. To elucidate the mechanisms underlying differential *nosZ* expression in *Bradyrhizobium* species, future studies need to incorporate comparative anal­yses across lineages and examine the interactions between *nosZ*‍ ‍and other denitrification genes. A more detailed understanding of these regulatory mechanisms will provide a basis for controlling *nosZ* expression and engineering efficient N_2_O-reducing bacteria.

## Citation

Wasai-Hara, S., Shimoda, Y., Mitsui, H., Sato, S., Imaizumi-Anraku, H., and Minamisawa, K. (2026) Promoter Sequences Do Not Solely Govern *nosZ* Expression Differences between *Bradyrhizobium ottawaense* and *B. diazoefficiens*. *Microbes Environ ***41**: ME25079.

https://doi.org/10.1264/jsme2.ME25079

## Supplementary Material

Supplementary Material

## Figures and Tables

**Fig. 1.**
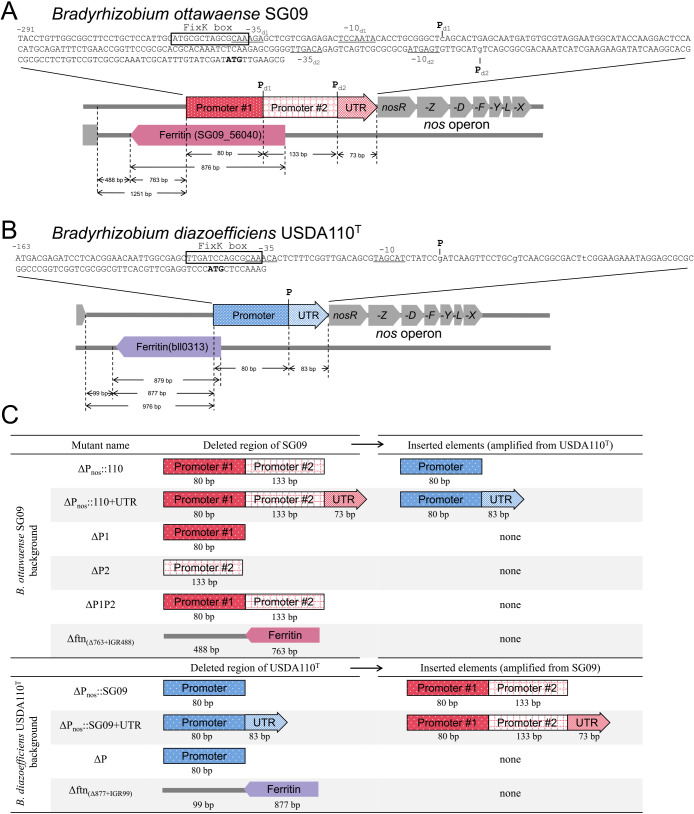
Promoter and upstream regions of the *nos* operon in *Bradyrhizobium ottawaense* SG09 and *B. diazoefficiens* USDA110^T^. (A, B) Upstream sequences and gene organization of the *nos* operon are shown. P_d1_, P_d2_, and P indicate the transcription start sites in SG09 and USDA110^T^. The –35 and –10 consensus sequences preceding each transcription start site are underlined. A putative FixK box is shown in the box. The translational start codon (ATG) of *nosR* is indicated in bold type. (A) In SG09, two promoters—the upstream region with promoter #1 (80 bp) and the downstream region with promoter #2 (133 bp)—followed by a 73-bp untranslated region (UTR), are arranged upstream of the *nos* operon in the 5′ direction. Upstream of these elements, a ferritin gene is located on the reverse strand. (B) In USDA110^T^, a single promoter and an 83-bp UTR are located upstream of the *nos* operon in the 5′ direction. Similarly, a ferritin gene is located on the reverse strand. (C) The design of the mutants constructed in the present study. The deleted region and inserted elements are shown.

**Fig. 2.**
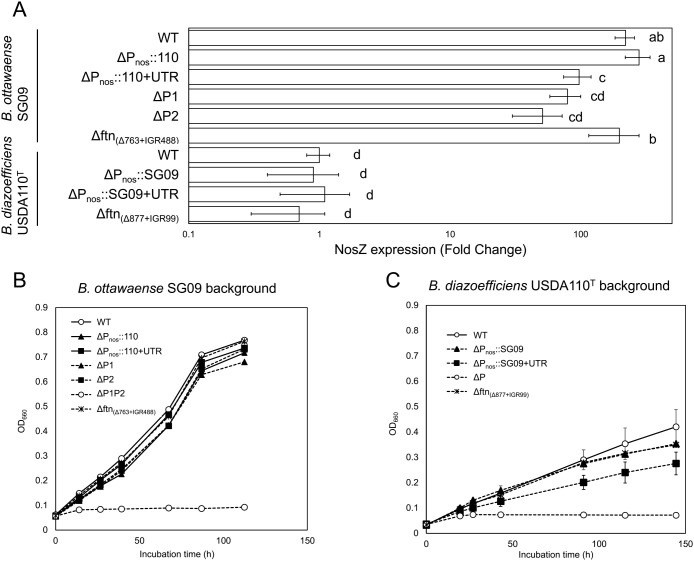
NosZ expression and growth rate in promoter-swapped strains under N_2_O-reducing conditions. (A) Relative *nosZ* transcript levels in *Bradyrhizobium ottawaense* SG09 background strains—wild-type (WT), promoter-swapped strain (ΔP_nos_::110), promoter and UTR-swapped strain (ΔP_nos_:110+UTR:), single promoter-deletion mutants (ΔP1 and ΔP2), and an upstream deletion mutant without promoter deletion (Δftn_[Δ763+IGR488]_)—as well as in *B. diazoefficiens* USDA110^T^ background strains—WT, promoter-swapped strain (ΔP_nos_::SG09), promoter and UTR-swapped strain (ΔP_nos_::SG09+UTR), and an upstream deletion mutant (Δftn_[Δ877+IGR99]_). Transcript levels were quantified by RT-qPCR and values are shown relative to the USDA110^T^ WT strain (set to 1). Bars represent means, and error bars indicate SD (*n*=4–6). Different letters above the bars represent significant differences between the inoculation treatments analyzed using Tukey’s test after an anal­ysis of variance (ANOVA; *P*<0.05). (B, C) Growth of SG09 and USDA110^T^ background strains under N_2_O-respiring conditions. Error bars indicate SD (*n*=3).

## References

[B1] Bhandari, B., and Nicholas, D.J.D. (1984) Denitrification of nitrate to nitrogen gas by washed cells of *Rhizobium japonicum* and by bacteroids from *Glycine max*. Planta 161: 81–85. 24253558 10.1007/BF00951463

[B2] Cole, M.A., and Elkan, G.H. (1973) Transmissible resistance to penicillin G, neomycin, and chloramphenicol in *Rhizobium japonicum*. Antimicrob. Antimicrob Agents Chemother 4: 248–253. 4491197 10.1128/aac.4.3.248 PMC444536

[B3] Gao, Y., Mania, D., Mousavi, S.A., Lycus, P., Arntzen, M.Ø., Woliy, K., et al. (2021) Competition for electrons favours N_2_O reduction in denitrifying *Bradyrhizobium* isolates. Environ Microbiol 23: 2244–2259. 33463871 10.1111/1462-2920.15404

[B4] Hallin, S., Philippot, L., Löffler, F.E., Sanford, R.A., and Jones, C.M. (2018) Genomics and ecology of novel N_2_O-reducing microorganisms. Trends Microbiol 26: 43–55. 28803698 10.1016/j.tim.2017.07.003

[B5] Hawley, D.K., and McClure, W.R. (1983) Compilation and anal­ysis of *Escherichia coli* promoter DNA sequences. Nucleic Acids Res 11: 2237–2255. 6344016 10.1093/nar/11.8.2237 PMC325881

[B6] He, G., Wang, W., Chen, G., Xie, Y., Parks, J.M., Davin, M.E., et al. (2025) A novel bacterial protein family that catalyses nitrous oxide reduction. Nature 646: 152–160. 40836093 10.1038/s41586-025-09401-4

[B7] Hiis, E.G., Vick, S.H.W., Molstad, L., Røsdal, K., Jonassen, K.R., Winiwarter, W., and Bakken, L.R. (2024) Unlocking bacterial potential to reduce farmland N_2_O emissions. Nature 630: 421–428. 38811724 10.1038/s41586-024-07464-3 PMC11168931

[B8] IPCC. (2023) *Climate Change 2023: Synthesis Report. Contribution of Working Groups I, II and III to the Sixth Assessment Report of the Intergovernmental Panel on Climate Change*. Core Writing Team, Lee, H., and Romero, J. (eds). Geneva, Switzerland: IPCC.

[B9] Itakura, M., Uchida, Y., Akiyama, H., Hoshino, T.Y., Shimomura, Y., Morimoto, S., et al. (2013) Mitigation of nitrous oxide emissions from soils by *Bradyrhizobium japonicum* inoculation. Nat Clim Change 3: 208–212.

[B10] Nishida, H., Itakura, M., Win, K.T., Li, F., Kakizaki, K., Suzuki, A., et al. (2025) Genetic design of soybean hosts and bradyrhizobial endosymbionts reduces N_2_O emissions from soybean rhizosphere. Nat Commun 16: 8023. 40908282 10.1038/s41467-025-63223-6 PMC12411642

[B11] Ravishankara, A.R., Daniel, J.S., and Portmann, R.W. (2009) Nitrous oxide (N_2_O): the dominant ozone-depleting substance emitted in the 21st century. Science 326: 123–125. 19713491 10.1126/science.1176985

[B12] Sameshima-Saito, R., Chiba, K., Hirayama, J., Itakura, M., Mitsui, H., Eda, S., and Minamisawa, K. (2006) Symbiotic *Bradyrhizobium japonicum* reduces N_2_O surrounding the soybean root system via nitrous oxide reductase. Appl Environ Microbiol 72: 2526–2532. 16597953 10.1128/AEM.72.4.2526-2532.2006 PMC1449076

[B13] Sánchez, C., Itakura, M., Okubo, T., Matsumoto, T., Yoshikawa, H., Gotoh, A., et al. (2014) The nitrate-sensing NasST system regulates nitrous oxide reductase and periplasmic nitrate reductase in *Bradyrhizobium japonicum*. Environ Microbiol 16: 3263–3274. 24947409 10.1111/1462-2920.12546

[B14] Sánchez, C., Mitsui, H., and Minamisawa, K. (2017) Regulation of nitrous oxide reductase genes by NasT-mediated transcription antitermination in *Bradyrhizobium diazoefficiens*. Environ Microbiol Rep 9: 389–396. 28474433 10.1111/1758-2229.12543

[B15] Sanford, R.A., Wagner, D.D., Wu, Q., Chee-Sanford, J.C., Thomas, S.H., Cruz-García, C., et al. (2012) Unexpected nondenitrifier nitrous oxide reductase gene diversity and abundance in soils. Proc Natl Acad Sci U S A 109: 19709–19714. 23150571 10.1073/pnas.1211238109 PMC3511753

[B16] Sciotti, M.A., Chanfon, A., Hennecke, H., and Fischer, H.M. (2003) Disparate oxygen responsiveness of two regulatory cascades that control expression of symbiotic genes in *Bradyrhizobium japonicum*. J Bacteriol 185: 5639–5642. 12949117 10.1128/JB.185.18.5639-5642.2003 PMC193759

[B17] Tian, H., Xu, R., Canadell, J.G., Thompson, R.L., Winiwarter, W., Suntharalingam, P., et al. (2020) A comprehensive quantification of global nitrous oxide sources and sinks. Nature 586: 248–256. 33028999 10.1038/s41586-020-2780-0

[B18] Torres, M.J., Bueno, E., Jiménez-Leiva, A., Cabrera, J.J., Bedmar, E.J., Mesa, S., and Delgado, M.J. (2017) FixK_2_ Is the main transcriptional activator of *Bradyrhizobium diazoefficiens nosRZDYFLX* genes in response to low oxygen. Front Microbiol 8: 1621. 28912756 10.3389/fmicb.2017.01621 PMC5582078

[B19] Wang, X., Xu, K., Tan, Y., Yu, S., Zhao, X., and Zhou, J. (2023) Deep learning-assisted design of novel promoters in *Escherichia coli*. Adv Genet 4: 2300184. 38099247 10.1002/ggn2.202300184 PMC10716054

[B20] Wasai-Hara, S., Hara, S., Morikawa, T., Sugawara, M., Takami, H., Yoneda, J., et al. (2020) Diversity of *Bradyrhizobium* in non-leguminous sorghum plants: *B. ottawaense* isolates unique in genes for N_2_O reductase and lack of the type VI secretion system. Microbes Environ 35: ME19102. 31932539 10.1264/jsme2.ME19102 PMC7104290

[B21] Wasai-Hara, S., Itakura, M., Fernandes Siqueira, A., Takemoto, D., Sugawara, M., Mitsui, H., et al. (2023) *Bradyrhizobium ottawaense* efficiently reduces nitrous oxide through high *nosZ* gene expression. Sci Rep 13: 18862. 37914789 10.1038/s41598-023-46019-w PMC10620151

[B22] Zhu, M., and Dai, X. (2025) Systematic modulation of bacterial resource allocation by perturbing RNA polymerase availability via synthetic transcriptional switches. Nucleic Acids Res 53: gkaf814. 40842238 10.1093/nar/gkaf814 PMC12370630

