# Supplementary material for: Promoter Sequences Do Not Solely Govern nosZ Expression Differences between Bradyrhizobium ottawaense and B. diazoefficiens
Source: Microbes Environ. 2026 Mar 3;41(1):ME25079. doi: 10.1264/jsme2.ME25079 (PMC12999732; doi:10.1264/jsme2.ME25079)
Supplement: Supplementary file 1 — Supplementary Material [file 41_25079_s1.pdf]

Promoter Sequences Do Not Solely Govern *nosZ* Expression Differences between *Bradyrhizobium ottawaense* and *B. diazoefficiens*

Supplemental information

Sawa Wasai-Hara[1, 2]*, Yoshikazu Shimoda[1], Hisayuki Mitsui[3], Shusei sato[3], Haruko Imaizumi-Anraku[1], Kiwamu Minamisawa[3]

[1]National Agriculture and Food Research Organization (NARO), Tsukuba, Ibaraki 305-8604, Japan
[2]National Institute of Advanced Industrial Science and Technology (AIST) Hokkaido, 2-17-2-1 Tsukisamu-higashi, Toyohira, Sapporo, Hokkaido 062-8517, Japan
[3]Graduate School of Life Sciences, Tohoku University, Katahira, Aoba-ku, Sendai 980-8577, Japan

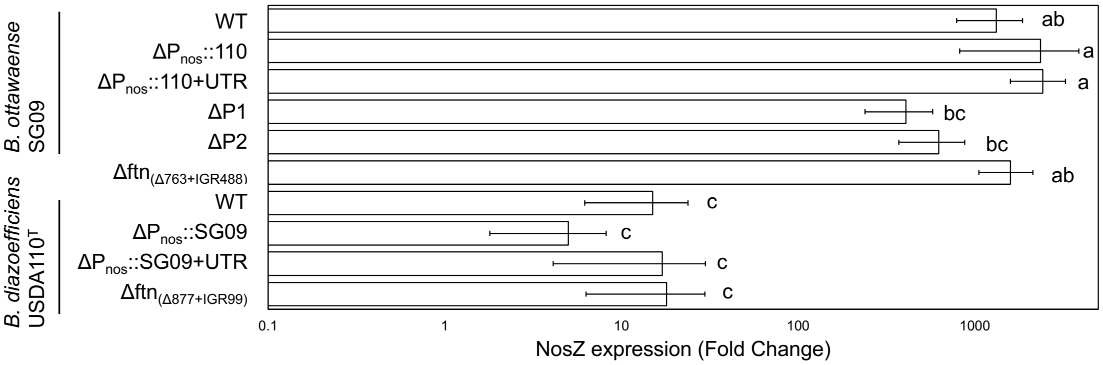

Fig. S1 *nosZ* expression and growth rate in promoter-swapped strains under NO_3^--reducing conditions.

Relative *nosZ* transcript levels in *Bradyrhizobium ottawaense* SG09 background strains—wild type (WT), promoter-swapped strain (ΔP_{nos}::110), promoter and UTR-swapped strain (ΔP_{nos}::110+UTR), single promoter-deletion mutants (ΔP1 and ΔP2), and upstream deletion mutant without promoter deletion ($\Delta ftn_{(\Delta 763+IGR488)}$)—as well as in *B. diazoefficiens* USDA110^T background strains—WT, promoter-swapped strain (ΔP_{nos}::SG09), promoter and UTR-swapped strain (ΔP_{nos}::SG09+UTR), and upstream deletion mutant ($\Delta ftn_{(\Delta 877+IGR99)}$). These strains were cultured in HM medium supplemented with 10 mM KNO_3. Transcript levels were quantified using RT-qPCR and normalized to the *sigA* gene. Values are shown relative to the USDA110^T WT strain (set to 1). Bars represent means, and error bars indicate SD (n = 5). Different letters above the bars represent significant differences between inoculation treatments analyzed using Tukey's test after analysis of variance (ANOVA; $p < 0.05$)

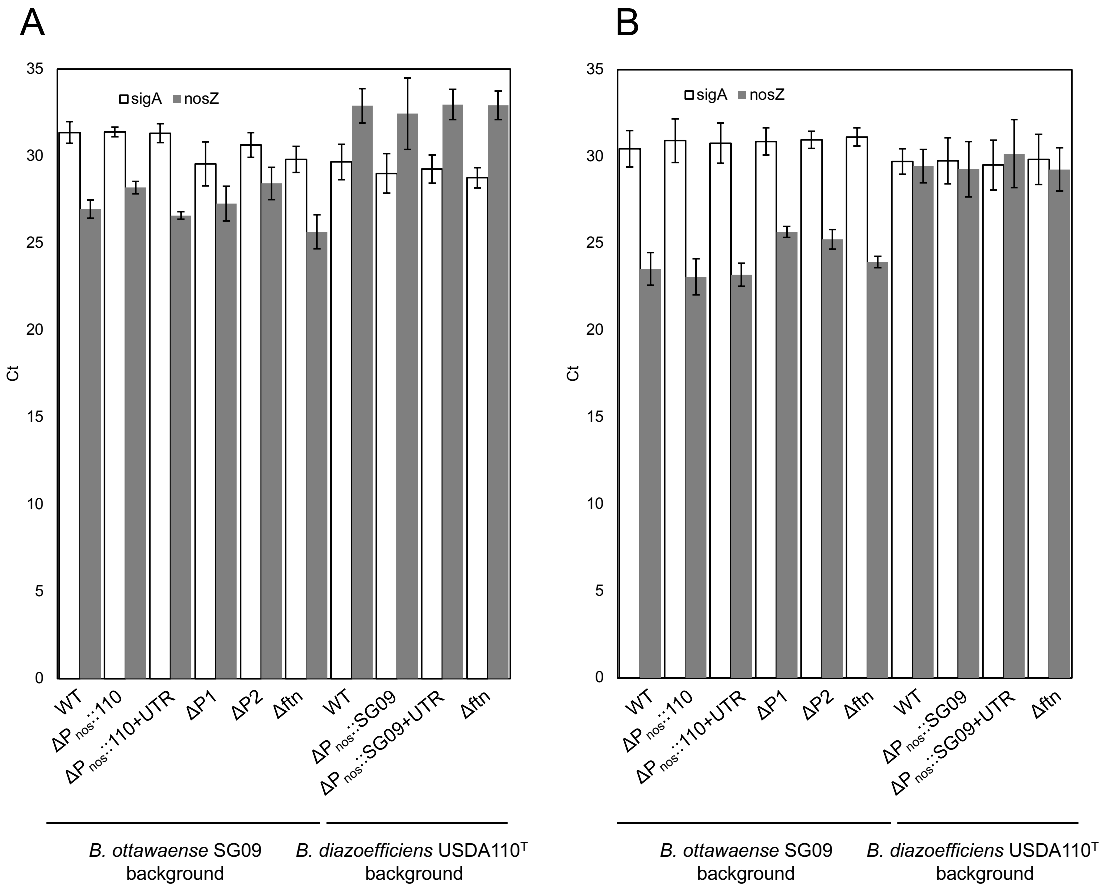

Fig. S2. Ct values in quantitative PCR.

(A) Ct values under N_2O-respiring conditions. Open bars indicate *sigA,* and filled bars indicate *nosZ*. Bars represent means, and error bars indicate SD (n = 4–6). These are the source data for Fig. 2A.

(B) Ct values under NO_3^--respiring conditions. Open bars indicate *sigA*, and filled bars indicate *nosZ*. Bars represent means, and error bars indicate SD (n =5). These are the source data for Fig. S1.

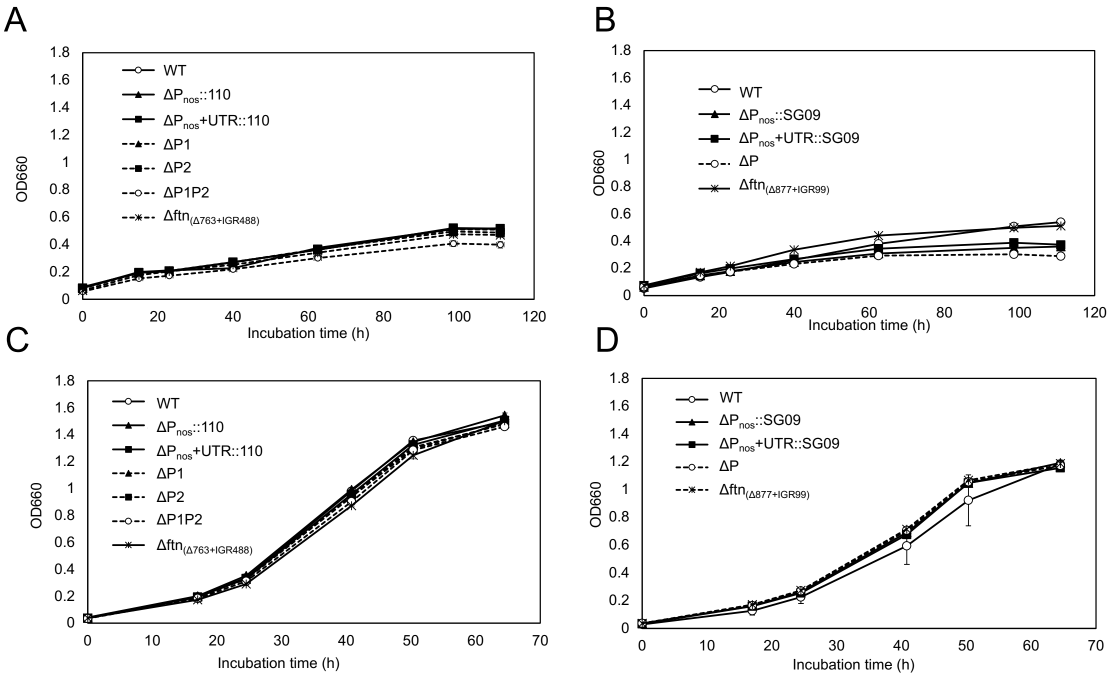

Fig. S3. Growth curves of promoter-swapped mutants of *B. ottawaense* SG09 and *B. diazoefficiens* USDA110^{T} under NO_3^--respiring and aerobic conditions.

Growth curves of mutants in the SG09 and USDA110^{T} backgrounds under (A, B) NO_3^--respiring and (C, D) aerobic conditions. OD_{660} was measured directly in 75 mL test tubes.

Table S1 Primers used in this study

Primer name	Sequence (5'→3')
ProU_del_F1	TCGAGCTCGGTACCCGTCAGATCTCGCCTGAGCTGAG
Pro1_2_del_R1	TCATCCGGTCGCTTCAACATCGAATCTCTCGCGCAAGACA
Pro1_2_del_F2	TGTCTTGCGCGAGAGATTCGATGTTGAAGCGACCGGATGA
ProU_del_R2	CTCTAGAGGATCCCCTTCAGCCGCTGATAGCCGTC
Pro_110_F	TGTCTTGCGCGAGAGATTCGATGACGAGATCCTCACGGAA
Pro_110_R	TCATCCGGTCGCTTCAACATGGGACCTCGAACGTGAACGC
Pro_del_110ins_R1	ATTGTTCCGTGAGGATCTCGTCATCGAATCTCTCGCGCAAGACA
Pro_del_110ins_F2	GCGTTCACGTTCGAGGTCCCATGTTGAAGCGACCGGATGA
Pro_del_09ATG_F2	CAAATCGCATTTGTATCGATATGCTCCAAAGAACGCATGTT
Pro110_09ATG_R	ATCGATACAAATGCGATTTGCGCGACGGACAGAGGCGCGCGTGCCTTGATATCTTCTTCGATGATTTGTCGCCGCTGACGGATAGATGCTACGCTGTCA
Pro110_del_F1	TCGAGCTCGGTACCCACGAACGACGTCGCATTCAAC
Pro110_del_R1	CAACATGCGTTCTTTGGAGCATGCCGTTGCTACGAACTTCGC
Pro110_del_F2	GCGAAGTTCGTAGCAACGGCATGCTCCAAAGAACGCATGTTG
Pro110_del_R2	CTCTAGAGGATCCCCGCCCCTTTGAAGGAATAACGG
Pro09_F	ACGAACGACGTCGCATTCAACTACCTGTTGGCGGCTTCCTG
Pro09_R	CAACATGCGTTCTTTGGAGCATATCGATACAAATGCGATTTGCGC
Pro_del_09ins_R1	CAGGAAGCCGCCAACAGGTAGCCGTTGCTACGAACTTCGC
Pro_del_09ins_F2	GCGCAAATCGCATTTGTATCGATATGCTCCAAAGAACGCATGTTG
Pro_del_110ATG_F2	GCGTTCACGTTCGAGGTCCCATGCTCCAAAGAACGCATGTTG
Pro09_110ATG_R	GGGACCTCGAACGTGAACGCCGCGACCGACCGGGCCGCGCGCTCCTATTTCTTCCGAAGTCGCCGTTGACGCAGGAACTTGATCATGCAACACTCATCGCGCG
Pro1_del_R1	CGCACATCATTGCTCAGTGCTCGAATCTCTCGCGCAAGACA
Pro1_del_F2	TGTCTTGCGCGAGAGATTCGAGCACTGAGCAATGATGTGCG
Pro2_del_R1	CGATGATTTGTCGCCGCTGAGAGCCCGCAGGTGTATTGGA
Pro2_del_F2	TCCAATACACCTGCGGGCTCTCAGCGGCGACAAATCATCG

Table S2 Primer combinations used for mutant construction

	Template	Target size (bp)
Promoter #1#2 deletion in SG09		
ProU_del_F1<br>Pro1_2_del_R1	SG09	900
Pro1_2_del_F2<br>ProU_del_R2	SG09	857
Promoter deletion in USDA110^T		
Pro110_del_F1<br>Pro110_del_R1	USDA110	900
Pro110_del_F2<br>Pro110_del_R2	USDA110	924
Insertion of USDA110^T promoter		
ProU_del_F1<br>Pro_del_110ins_R1	SG09	900
Pro_del_110ins_F2<br>ProU_del_R2	SG09	935
Pro_110_F<br>Pro_110_R	USDA110^T	94
Insertion of USDA110^T promoter and UTR		
ProU_del_F1<br>Pro_del_110ins_R1	SG09	900
Pro_del_09ATG_F2<br>ProU_del_R2	SG09	935
Pro110_F<br>Pro110_09ATG_R	USDA110^T	172
Insertion of SG09 promoter		
Pro110_del_F1<br>Pro_del_09ins_R1	USDA110^T	900
Pro_del_09ins_F2<br>Pro110_del_R2	USDA110^T	924
Pro09_F<br>Pro09_R	SG09	213
Insertion of SG09 promoter and UTR		
Pro110_del_F1<br>Pro_del_09ins_R1	USDA110^T	900
Pro_del_09ATG_F2<br>Pro110_del_R2	USDA110^T	924
Pro09_F<br>Pro09_110ATG_R	SG09	293
Deletion of SG09 promoter#1		
ProU_del_F1<br>Pro1_del_R1	SG09	900
Pro1_del_F2<br>ProU_del_R2	SG09	1068
Deletion of SG09 promoter#2		
ProU_del_F1<br>Pro2_del_R1	SG09	980
Pro2_del_F2<br>ProU_del_R2	SG09	935
